# An Exploratory Approach to Deriving Nutrition Information of Restaurant Food from Crowdsourced Food Images: Case of Hartford

**DOI:** 10.3390/nu13114132

**Published:** 2021-11-18

**Authors:** Xiang Chen, Evelyn Johnson, Aditya Kulkarni, Caiwen Ding, Natalie Ranelli, Yanyan Chen, Ran Xu

**Affiliations:** 1 Department of Geography, University of Connecticut, Storrs, CT 06269, USA; xiang.chen@uconn.edu; 2 Department of Allied Health Sciences, University of Connecticut, Storrs, CT 06269, USA; evelyn.johnson@uconn.edu (E.J.); natalie.ranelli@uconn.edu (N.R.); yanyan.chen@uconn.edu (Y.C.); 3 Department of Computer Science & Engineering, University of Connecticut, Storrs, CT 06269, USA; aditya.kulkarni@uconn.edu (A.K.); caiwen.ding@uconn.edu (C.D.)

**Keywords:** nutrition assessment, food image, image recognition, restaurant, food environment, FAFH, crowdsourcing, deep learning, GIS, Hartford

## Abstract

Deep learning models can recognize the food item in an image and derive their nutrition information, including calories, macronutrients (carbohydrates, fats, and proteins), and micronutrients (vitamins and minerals). This technology has yet to be implemented for the nutrition assessment of restaurant food. In this paper, we crowdsource 15,908 food images of 470 restaurants in the Greater Hartford region on Tripadvisor and Google Place. These food images are loaded into a proprietary deep learning model (Calorie Mama) for nutrition assessment. We employ manual coding to validate the model accuracy based on the Food and Nutrient Database for Dietary Studies. The derived nutrition information is visualized at both the restaurant level and the census tract level. The deep learning model achieves 75.1% accuracy when compared with manual coding. It has more accurate labels for ethnic foods but cannot identify portion sizes, certain food items (e.g., specialty burgers and salads), and multiple food items in an image. The restaurant nutrition (RN) index is further proposed based on the derived nutrition information. By identifying the nutrition information of restaurant food through crowdsourced food images and a deep learning model, the study provides a pilot approach for large-scale nutrition assessment of the community food environment.

## 1. Introduction

Americans’ eating habits have been through a drastic change—they are spending more on eating out rather than cooking at home [1,2]. According to the United States (US) Department of Agriculture (USDA) Economic Research Service Food Expenditure Series, the total sales of food prepared away from home (FAFH) surpassed that prepared at home (FAH) for the first time in 2014 [3]. The gap between the two expenditures continued to widen over the last few years. In 2019, the expenditures for FAFH were approximately $389,677 million, while the total expenditure for FAFH exceeded $418,933 million [3]. The largest portion of the FAFH (i.e., 36.8% based on the 2019 Food Expenditure Series [3]) was consumed at a limited-service restaurant, which is generally known as a fast-food restaurant. Compared to FAH, FAFH is relatively calorie-dense and nutrient-poor, as it contains more saturated fat, sodium, and cholesterol but less dietary fiber [4]. Thus, the change in dietary behaviors has posed risks for FAFH consumers to develop obesity and obesity-related chronic diseases (e.g., Type II diabetes and cardiovascular diseases). For example, recent literature identified a strong association between FAFH consumption and calorific intake among children, which strengthened the evidence of the health adversities as a result of FAFH consumption [5,6].

To evaluate the nutrition of FAFH, it is essential to employ nutrition assessment methods on individual diets. This evaluation normally takes two different approaches. The first approach refers to the dietary assessment using nutritional biomarkers. Nutritional biomarkers are clinical instruments to identify the existence of nutrients in biological samples and thus can be used as a proxy for the human body’s nutrient absorption and metabolic response to food consumption [7,8]. While nutritional biomarkers can objectively quantify the nutritional status of samples, their employment is equipment-dependent and restricted to clinical settings. Additionally, the evaluation results are subject to an individual’s disease status or homeostatic regulation [9]. The second approach refers to the individual dietary assessment, such as the food frequency questionnaire (FFQ), 24-h dietary recall (24HR), and dietary record (DR). This approach evaluates individuals’ food consumption and dietary patterns by structured surveys or in-depth interviews. Although the individual dietary assessment is a direct observation of food consumption patterns and can be implemented in non-clinical settings, it is subject to systematic biases induced by human subjects, including recall error and reporting bias [10]. Another prevailing issue in both approaches is that they require considerable efforts in data collection and processing, including personnel training, sample testing, interviewing, and data coding. For these reasons, these traditional nutrition assessment methods cannot be easily implemented on a large scale.

Advances in food image capturing and recognition technologies provide alternative means to dietary data collection and nutrition assessment. Food image recognition was initially explored in a pilot study by Williamson et al. [11], which employed digital photography and visual estimation for food selections, plate waste, and portion sizes. This method, further coined as the Remote Food Photography Method (RFPM) [12], was reaffirmed by cross-validating the estimated nutrients in food photographs with trained raters [12] and nutritional biomarkers [13]. The development and advancements in mobile devices and internet services further popularized the use of this technology, allowing individuals to record their dietary intake. For example, a prototype mobile device, called Wellnavi, was used in capturing dietary data for clinical assessments and dietary interventions [14]. In another case, the captured food images were cross-validated with individuals’ voices to verbally describe food items [15]. However, mobile devices in these initial attempts served only as instruments for data collection, storage, and transfer. The actual nutrition assessment component was still reliant on traditional measures, such as FFQ, 24HR, and DR [10]. More recently, technological advancements in computer science provide new opportunities for leveraging dietary data for effective nutrition assessment. These computer-aided methods, primarily deep learning models, can identify the actual food item in an image [16,17,18]. Meanwhile, complexities in food images, such as portion sizes [19] and the co-existence of multiple food items [20], were also resolved by deep learning algorithms. In addition, proprietary mobile apps were developed to estimate the nutrition facts of the food in an image through cloud services, where a nutrient composition database is hosted. Examples of these cutting-edge food image recognition apps (where nutrition facts can be simultaneously estimated) include Calorie Mama, Foodzilla, Lose it!, and Mealviser.

While food image recognition by deep learning models has the potential to inform dietary decisions and facilitate health promotion, to date, this method has not been applied on a large scale (e.g., all restaurants in a city). In this paper, we investigate the applicability of this technology for the nutrition assessment of restaurant food using crowdsourced food review images in a US metropolitan area, the Greater Hartford region. Then, we validate the deep-learned results with manually coded nutrition information. Lastly, the paper explores the implication of the new method for community food environmental studies through nutrition mapping and food inequality assessment. These endeavors not only exhibit an exploratory case study of the deep learning model for nutrition assessment but also the potential of the new method for assisting health policymaking and health promotion.

## 2. Materials and Methods

Our case study was conducted in the Greater Hartford region in the US. As the capital city of Connecticut, Hartford is the fourth most populous city in the state. The total population of Hartford County was estimated to be 891,720 as of 2019, where the demographics (74.8% white, 15.8% African American, 6.1% Asian, 0.6% Native American) were close to the national average [21]. Because of its vibrant economy and diverse populations, the city serves as a cultural destination and food hub for Central and Eastern Connecticut. As Hartford has intensive spatial interaction with surrounding areas in terms of traffic, human movements, and services, we expanded our study area to Hartford and its five satellite cities (Bloomfield, West Hartford, East Hartford, Newington, and Wethersfield) to portray a comprehensive foodscape.

### 2.1. Data Collection

**Figure 1:**
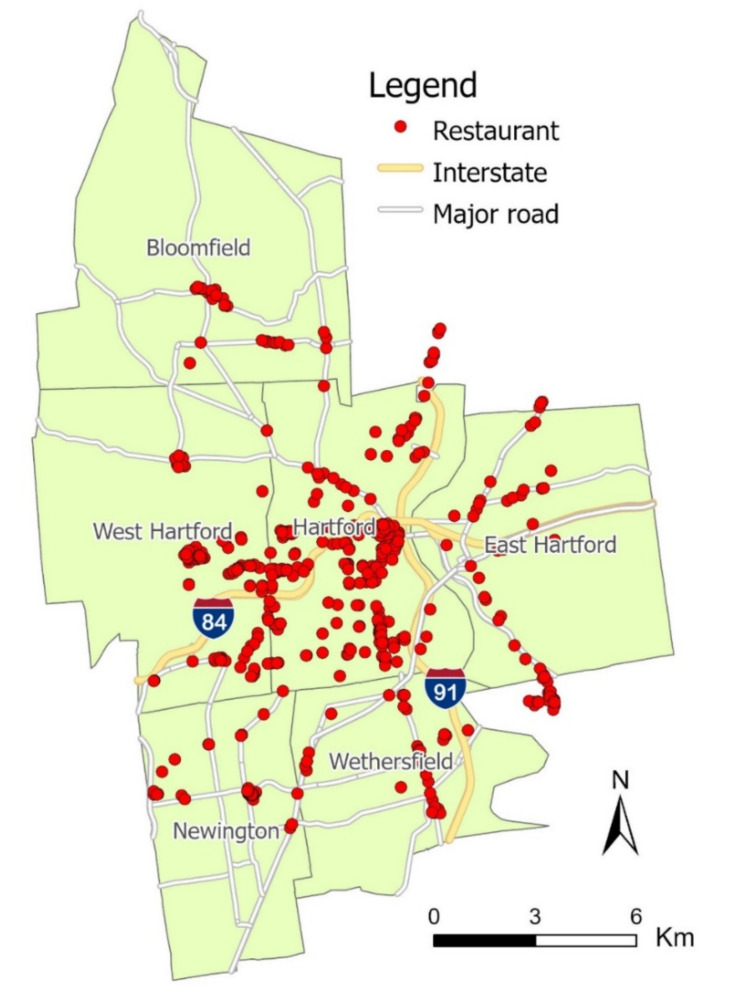
Spatial distribution of restaurants in the study area.

As the study was focused on the nutrition assessment of restaurant food, we utilized two datasets: the restaurant directory and food review images. The first dataset, the restaurant directory, included the business information (e.g., name, address, hours of operation, and contact information) of all restaurants in the study area. This dataset was sourced by using Yelp (i.e., a business listing website with foci on restaurant ratings and reviews) and its Fusion application programming interface (API) [22]. The data were further processed by Python codes to retrieve additional information, such as restaurant category, rating, and review count. Then, we cleaned the data by manually cross-validating with Google Place (i.e., a business listing website with foci on restaurant locations and reviews), purging restaurants that were unidentified, permanently closed, or mislabeled (e.g., supermarkets, convenience stores, food pantries). This cross-validation process using Yelp and Google Place ensured a high degree of accuracy and timeliness in the restaurant directory. Eventually, 487 restaurants fit the inclusion criteria and composed the initial restaurant directory dataset for further investigation. These restaurants were visualized in ESRI ArcGIS Pro, as shown in Figure 1.

Our second dataset consisted of food review images shared by online users. These images were collected from two different sources—Google Place and Tripadvisor (i.e., a travel advisory website with user-generated reviews)—and were combined for the same restaurant listing. Specifically, the image data collection was conducted by using the simple mass downloader extension on Google Chrome [23]. A total of 19,907 images were initially collected and were manually refined against the following exclusion criteria: (1) the image was staged or was part of an advertisement; (2) the image featured beverages; (3) the image was about a non-food item, such as buildings, dining environments, and people; (4) the restaurant had less than five images. The data collection from two sources and the follow-up filtering process ensured a high degree of completeness and accuracy in the food image dataset. Our final food image dataset included 15,908 images from 470 restaurants, where each image was related to the restaurant by a common ID. These food images were further standardized to 544 by 544 pixels by Python scripting for further processing in the deep learning model.

It should be noted that as of 2021, Google, Yelp, and Tripadvisor were the top three review platforms for consumers to make decisions about business patronage [24]. Thus, the choice of the three platforms in our study in terms of retrieving the restaurant directory, crowdsourcing food images, and cross-validation ensured the representativeness of the data and avoided the possible selection bias of just focusing on one platform.

### 2.2. Nutrition Assessments by Deep Learning Model and Manual Coding

**Figure 2:**
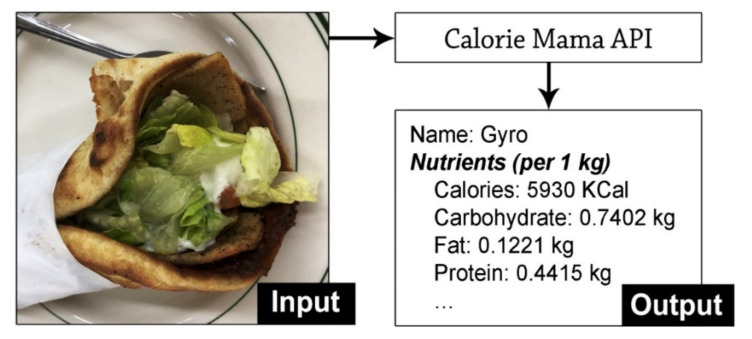
Example of nutrition assessment by the deep learning model.

We employed a proprietary deep learning model, Calorie Mama [25], for the nutrition assessment of food images. Developed by Azumio Inc. (Palo Alto, US), Calorie Mama is a deep learning-based image recognition model aiming at the nutrition assessment of food images. We chose Calorie Mama, as a recent comparative study showed that Calorie Mama was the most accurate platform with a top 1 accuracy of 63% and a top 5 accuracy of 88% [26]. When a food image is loaded into the deep-learning model, the model returns the most likely food label and its corresponding nutrition information. The derived nutrition information includes calories, macronutrients (carbohydrates, fats, and proteins), and micronutrients (vitamins and minerals) in the International System of Units (SI) (e.g., calories per 1 kg food). In addition, the model can identify not only fresh produce but also prepared dishes, including regional cuisines and ethnic specialty dishes. Figure 2 shows an example of the nutrition assessment for a crowdsourced food image.

We employed the Calorie Mama API to batch process all collected food images and then derived their nutrition information. To validate the results, two trained raters performed a manual nutrition assessment on a random sample of 281 images from 20 restaurants. The two raters independently coded the sample, including food type, nutrition information, and portion size, based on the 2017–2018 Food and Nutrient Database for Dietary Studies (FNDDS) [27]. Out of the 281 images, 75 images were double-coded to examine the inter-rater reliability and ensure the validity of the assessment. The rated nutrition information was then compared with that identified by the deep learning model.

### 2.3. Restaurant Nutrition (RN) Index

**Figure 3:**
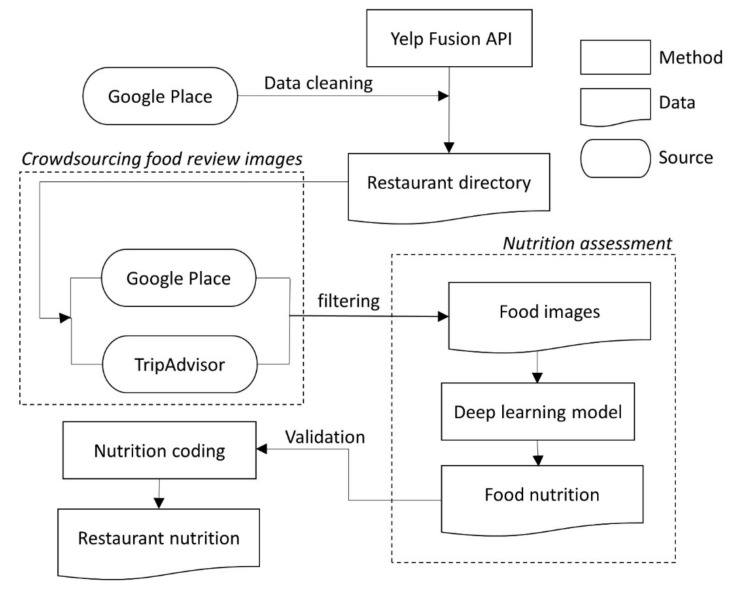
Flowchart of nutrition assessment by the deep learning model.

Lastly, we performed the nutrition assessment of the restaurants based on the average calories of all food images for each restaurant (i.e., average calories per 1 kg food) and visualized the nutrition information in a Geographic Information System (GIS). Furthermore, we proposed the restaurant nutrition (RN) index by aggregating the restaurants’ calorie estimates on the census tract level. We validated this new index by (1) quantitatively comparing it with an established food environment index—the Modified Retail Food Environment Index (mRFEI) [28], which evaluates the ratio of healthy food retailers (e.g., supermarkets) to unhealthy food retailers (e.g., fast-food restaurants) in a census tract, and (2) assessing the Pearson’s correlations between the RN index and key variables derived from the CDC’s 2018 Social Vulnerability Index (SVI). The SVI utilizes American Community Survey’s (ACS) 5-year estimates to determine the relative vulnerability of census tracts under four categories: socioeconomic status (SES), household composition and disability, minority status and language, and housing type and transportation [29]. The flow chart of the study is shown in Figure 3.

## 3. Results

### 3.1. Deep Learning Model Validation

Out of the 75 images that were double-coded, the two raters agreed with each other on 71 images in terms of food types and FNDDS codes, reaching inter-rater reliability of 94.7%. Out of the 281 coded images, we found that the deep learning model correctly identified 211 food images, reaching an accuracy level of 75.1%. Four images were incorrectly identified by both the manual coding and the deep learning model due to poor image quality. It is noted that the deep learning model had more specific and accurate food labels for 24 images, which were mostly ethnic food items (i.e., specifically for Korean dishes and less so for Mexican, Italian, and Chinese dishes). These ethnic food labels were not specified in the FNDDS.

The deep learning model is subject to various limitations. First, we found that the model had inaccurate identifications for images containing multiple food items, where it could only identify one of the food items present in the image. Second, the identification of certain food items was less precise and accurate. For example, the model identified most sandwiches and burgers as the “beef burger”; it also labeled many specialty salads as the “Caesar salad”, while there was apparent variability in the salad type by manual coding. Third, the deep learning model was unable to estimate the portion size from a food image.

### 3.2. Nutrition Mapping

**Figure 4:**
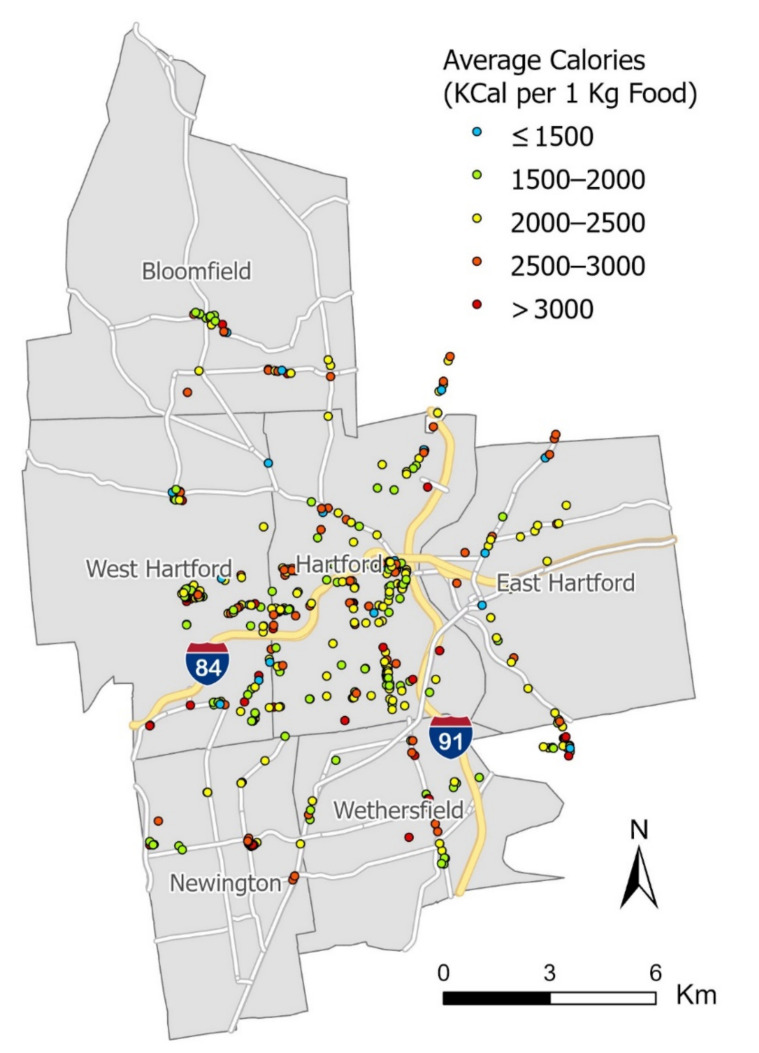
Estimated calorific level of restaurants in the study area.

The nutrition information identified from the 15,908 images for 470 restaurants can be further employed to estimate the nutrition quality of restaurants, which cannot be easily accomplished by traditional nutrition assessment methods. In this study, we estimated the nutrition quality of a restaurant by averaging the calories for all of its food images in the normalized SI unit (i.e., average calories for 1 kg food). Since the nutrition information is standardized on the restaurant level, it can be compared across all restaurants. We then employed ESRI ArcGIS Pro to map the calorific level in five color-coded classes, where the blue dots represent the lowest-calorie level and the red dots the highest. The mapping result is shown in Figure 4.

### 3.3. Restaurant Nutrition Index—Measuring the Community Food Environment

**Figure 5:**
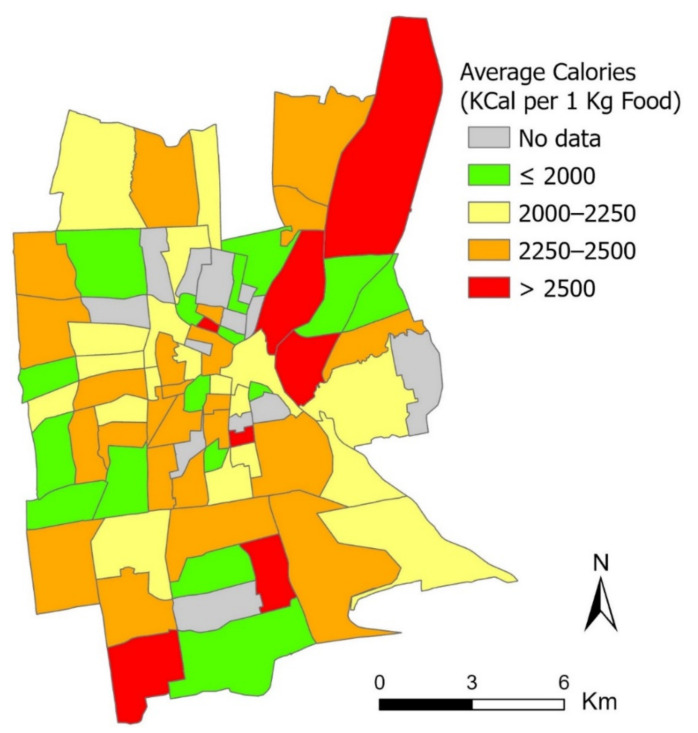
The RN index in terms of average calories of restaurant food by census tract.

The derived nutrition information on the restaurant level can be further leveraged for justifying critical inequality issues in the community food environment. This revelation can contribute to the lack of nutrition component in existing community food environmental studies [30]. Specifically, we have proposed a restaurant nutrition (RN) index by aggregating the restaurants’ calorie estimates for each census tract (mean = 2214.92, standard deviation (SD) = 276.98, min = 1500, max = 3028.88). The result is shown in Figure 5. This result reveals that the high-index census tracts (red-colored tracts in Figure 5), representing areas with the relative concentration of high-calorie restaurants, are mostly found in the northeast quadrant of the study area, which also happens to be the areas with low food access and fewer healthy food retailers as measured by other food environment indices, such as the Food Access Research Atlas [31] and the mRFEI. While the low-index census tracts (green-colored tracts in Figure 5) are more scattered geographically, a moderate consistency is identified between the RN index and the mRFEI in some census tracts, especially in the western and southern quadrants of the study area. Overall, we have identified a moderate consistency and weak Pearson’s correlation between the RN index and the mRFEI (*r* = −141, *p* = 0.26).

To further explore the inequality patterns in the nutrition landscape, we correlated the derived RN index with selected socioeconomic and demographic variables in CDC’s 2018 SVI data on the census tract level [29]. The Pearson’s correlation analysis was performed between selected SVI variables with the RN index across census tracts with available data (*n* = 66). The result is shown in Table 1.

Table 1 shows that there are moderate positive correlations between the RN index and three SVI variables representing socioeconomic status, household composition, and housing type, respectively. These correlated variables include % persons (age 25+) with no high school diploma (*r* = 0.24, *p* = 0.057), % single-parent household with children (*r* = 0.29, *p* = 0.018), and % persons in group quarters (*r* = 0.37, *p* = 0.002). The result signifies that the food inequality pattern in terms of restaurant nutrition does not have a perfect one-to-one matching with social vulnerability in the study area, as the RN index only correlates with some social vulnerability indicators but not the others (e.g., poverty rate, income, and vehicle access). The result can be explained by the argument of using fast-food access as a deprivation indicator—a systematic literature review shows that while many studies (i.e., 16 out of 21 studies) identified that fast-food restaurants were more prevalent in SES-deprived areas, other studies did not reveal such a correlation [32]. To this end, our study can shed insights into the justification of the food inequality—although the Greater Hartford region is regarded as one of the most segregated US metropolitan areas [33], access to nutritional restaurants across different neighborhoods may be less segregated and exhibit a complex geographical pattern.

## 4. Discussion

While deep learning models have been vigorously developed for food image recognition and nutrition analysis, this study is among the first to leverage this emerging technology for large-scale nutrition assessment of restaurant food. By crowdsourcing food images from food review websites, the study provides a pilot approach to restaurant nutrition assessment and can complement traditional nutrition assessment measures.

First, the study is among the first to bridge a crowdsourcing approach with a deep learning model to improve the efficiency of nutrition assessment. Existing nutrition assessment measures, including individual dietary assessments (e.g., FFQ, 24HR, and DR) [10] and nutrition environment assessments (e.g., Nutrition Environment Measure Survey—Restaurant [NEMS-R]) [34], collect data on the individual or restaurant level. While they standardize the protocol and variables in the assessment, they are subject to considerable efforts in data collection, testing, and coding. The crowdsourcing method applied to all restaurants on a large scale can automate dietary data collection by largely reducing time, labor, and cost. Additionally, we validated the accuracy of the deep learning model at 75.1% based on the FNDDS codes. Although the accuracy level was not as high as those of the traditional measures, the new method can serve to gain an overarching picture of the regional restaurants’ nutrition landscape at a relatively low cost and high efficiency. From a practical perspective, this method is easily scalable and can be implemented for the nutrition assessment of small, individual-owned restaurants or in underdeveloped countries where nutrition labeling is not readily available.

Second, mapping the nutritional information of restaurants can shed insights into health policymaking and health promotion. With advances in geospatial technologies, primarily GIS, it has become viable to reveal the spatial distribution of food sources across communities [35] and develop food indices and tools, such as the Food Access Research Atlas [31], the Food Environment Atlas [36], the “food swamp” index [37,38], and the mRFEI [28]. These spatial endeavors have been criticized for an overemphasis on the spatial pattern of food establishments (e.g., proximity, density, varieties), while lacking the food quality and nutrition measures to justify the food environment–diet relationship [39,40]. The quality and nutrition of food sources, coined by Glanz et al. [30] as the consumer nutrition environment, play a pivotal role in dictating community health. Our study takes a major step in filling this gap by empirically measuring food quality and nutrition in this consumer nutrition environment. While our results show that restaurants with higher calories are more likely to locate in socially vulnerable areas to some degree (e.g., population with lower education, more single-parent households, and living in group quarters), the weak correlation between our proposed RN index and the mRFEI is also somewhat expected and points to the potential discrepancies between spatial food provisioning and food nutrition. By revealing the inequality of nutrition information across different communities, stakeholders can go beyond the simple categorization of food sources and be informed of regional pockets where calorie-dense, nutrient-poor restaurants prevail and where efforts for nutrition assistance and improvement should prioritize. Moreover, the nutrition mapping results can be further developed into an interactive tool to facilitate health promotion in communities inundated with calorie-dense, nutrient-poor restaurants.

As a pilot study, this research has limitations. First, using the crowdsourcing approach deviated from a systematic sampling method and might not fully characterize all restaurants’ nutrition information. The primary dataset, the food images, was solicited from two food review websites. This crowdsourcing approach excluded nutrition information from restaurants that lacked an online presence. For example, it was found that restaurant reviews on Google Place were unevenly distributed, where national chain restaurants were less likely to receive a review than independently-operated restaurants [41]. Second, there were considerable uncertainties about the users who uploaded the food images, as the restaurant reviewers did not mirror the demographics of local residents because of the existence of the “digital divide” [42]. Specifically, young adults were overrepresented in the online community [43] and could influence the restaurants and the food items being reviewed. Third, our tests of validity showed that the deep learning model achieved only 75.1% accuracy, as the model was incapable of estimating portion sizes, identifying certain food items, or distinguishing multiple food items in an image. It is expected that the model performance can be improved by incorporating other food image recognition methods [16,17,18] and by cross-validating the results with trained raters. Finally, we only focused on the Greater Hartford region on the census tract level, and therefore the correlation results may not be generalized for another study area or on a different analysis scale. However, the study design and implementation are transferrable to other study areas, especially in countries where menu labeling data are missing.

## 5. Conclusions

In this paper, we explore a new deep learning approach for the nutrition assessment of restaurant food. We also validate the accuracy of the method by cross-validating with the FNDDS calorie information. We further estimate the nutrition information of restaurants in the study area and further develop the RN index to explore the food inequality issue in the consumer nutrition environment. Our results show that the deep learning model can be empowered by the crowdsourced food images through gathering dietary data at a minimum cost and acceptable data quality. However, the new method is still in the early phase of development due to the compromised accuracy in the model performance and the many uncertainties in the user-generated dietary data. Thus, this new method should only complement, rather than replace, traditional nutrition assessment methods for estimating the nutrition information. We believe that the new method holds promise as a new instrument for large-scale nutrition assessment when the deep learning model is further improved and when additional means are employed for data screening and result validation. Eventually, we expect that the deep-learned nutrition information can serve as evidence for developing an information system for nutrition education and health promotion.

## Figures and Tables

**Table 1:** Pearson’s correlation analysis between selected SVI variables and the RN index on the census tract level (*n* = 66).

SVI Variable	Mean (SD)	Min/Max	Correlation Coefficient (R)
Persons (%) below poverty	17.44 (13.38)	0/49.2	0.03
Unemployment rate (%)	9.16 (5.70)	0/23.3	−0.04
Per capita income	32,691.94 (16646.26)	5509/68,705	−0.18
Persons (%, age 25+) with no high school diploma	17.19 (12.65)	0.3/49	0.24 *
Persons (%) aged 65 and older	14.76 (6.32)	1.6/28.7	−0.06
Persons (%) aged 17 and younger	21.54 (6.77)	2.1/39.3	−0.21 *
Persons (%) with a disability	13.02 (4.43)	0/25.4	−0.03
Single-parent household (%) with children	13.88 (14.19)	0.5/100	0.29 **
Minority (%)	60.95 (30.79)	9/100	−0.01
Persons (%, age 5+) who speaks english less than well	7.44 (6.80)	0/25.1	0.04
Housing structures (%) with 10 or more units	20.29 (20.56)	0/87.3	−0.09
Mobile homes (%)	0.78 (3.60)	0/25.5	0.15
Occupied housing units (%) with more people than rooms estimate	3.02 (2.96)	0/10.3	−0.05
Households (%) with no vehicle	18.89 (14.55)	0/60.4	−0.03
Persons (%) in group quarters	3.94 (12.38)	0/93.4	0.37 ***

*** *p* < 0.01, ** *p* < 0.05, * *p* < 0.1. SVI variables with *p* < 0.05 are in bold.

## Data Availability

The data presented in this study are available upon request from the corresponding author. The data are not publicly available due to privacy concerns.
